# Is Obesity a Risk Factor for Carotid Atherosclerotic Disease?—Opportunistic Review

**DOI:** 10.3390/jcdd9050162

**Published:** 2022-05-17

**Authors:** Joana Ferreira, Pedro Cunha, Alexandre Carneiro, Isabel Vila, Cristina Cunha, Cristina Silva, Adhemar Longatto-Filho, Amílcar Mesquita, Jorge Cotter, Margarida Correia-Neves, Armando Mansilha

**Affiliations:** 1 Vascular Surgery Department, Hospital de Trás-os-Montes, 5000-508 Vila Real, Portugal; 2 Life and Health Science Research Institute (ICVS), School of Medicine, University of Minho, 4710-057 Braga, Portugal; pedrocunha@hospitaldeguimaraes.min-saude.pt (P.C.); isabelvila@hospitaldeguimaraes.min-saude.pt (I.V.); cristina.i.cunha@gmail.com (C.C.); cristinasmsilva@gmail.com (C.S.); longatto@med.uminho.pt (A.L.-F.); jorgecotter@gmail.com (J.C.); mcorreianeves@med.uminho.pt (M.C.-N.); 3 Center for the Research and Treatment of Arterial Hypertension and Cardiovascular Risk, Internal Medicine Department, Hospital da Senhora da Oliveira, 4835-044 Guimarães, Portugal; 4 Medicine Department, Hospital da Senhora da Oliveira, 4835-044 Guimarães, Portugal; 5 Radiology Department, Unidade Local de Saúde Alto Minho, 4904-858 Viana do Castelo, Portugal; alexandrelimacarneiro@gmail.com; 6 Department of Pathology (LIM-14), University of São Paulo School of Medicine, São Paulo 01246-903, Brazil; 7 Molecular Oncology Research Center, Barretos Cancer Hospital, São Paulo 14784-400, Brazil; 8 Vascular Surgery Department, Hospital da Senhora da Oliveira, 4835-044 Guimarães, Portugal; amilcarmesquita@hospitaldeguimaraes.min-saude.pt; 9 Faculdade de Medicina da Universidade do Porto, 4200-319 Porto, Portugal; vascular.mansilha@gmail.com; 10 Vascular Surgery Department, Hospital de São João, 4200-319 Porto, Portugal

**Keywords:** obesity, visceral adipose tissue, subcutaneous adipose tissue, carotid atherosclerotic disease

## Abstract

Obesity is a risk factor for coronary atherosclerosis. However, the influence of adipose tissue in carotid atherosclerosis is not completely understood. No systematic review/meta-analysis was previously performed to understand if obesity is a risk factor for carotid atherosclerosis. This paper aims to provide an opportunistic review of the association between obesity and carotid atherosclerosis and define the role of the different adipose tissue depots in the characteristics of carotid stenosis. The databases PubMed and Cochrane Library were searched on 15–27 April and 19 May 2021. A total of 1750 articles published between 1985 and 2019 were identified, 64 were preselected, and 38 papers (35,339 subjects) were included in the final review. The most frequent methods used to determine obesity were anthropometric measures. Carotid plaque was mostly characterized by ultrasound. Overall obesity and visceral fat were not associated with the presence of carotid plaque when evaluated separately. Waist-hip ratio, however, was a significant anthropometric measure associated with the prevalence of carotid plaques. As it reflected the ratio of visceral and subcutaneous adipose tissue, the balance between these depots could impact the prevalence of carotid plaques.

## 1. Introduction

The worldwide prevalence of obesity has increased in the past decades. Obesity is an independent risk factor for atherosclerosis, stroke, and cardiovascular disease [1,2,3]. Cardiovascular outcomes and mortality are more dependent on fat distribution than on the total amount of adipose tissue [1]. Previous studies demonstrated that a higher ratio of visceral adipose tissue (VAT) to subcutaneous adipose tissue (SAT) was associated with an increased risk of poor cardiovascular outcomes [1]. VAT is metabolically active and secret adipokines that cause vascular inflammation and insulin resistance [2]. Conversely, SAT is associated with a neutral or even beneficial metabolic impact [2].

Increased abdominal VAT/SAT ratio was inversely correlated with the extent and severity of coronary artery plaques, higher total mortality, and incidence of major adverse cardiac events (MACE) [1,2].

The influence of obesity on carotid atherosclerosis disease remains unclear [4]. No previous systematic review/metanalyses evaluated the relationship between obesity on carotid atherosclerosis.

## 2. Methods

### 2.1. Data Sources and Search

The PubMed and Cochrane Library databases were searched on 15–27th of April and 19 May 2021. The query was as follows:

(‘Carotid artery stenosis OR ‘Carotid artery atherosclerosis’) AND (‘visceral adipose tissue’ OR ‘visceral fat’ OR ‘fat tissue’ OR ‘Obesity’ OR ‘subcutaneous adipose tissue’ OR ‘subcutaneous fat’).

This review was conducted according to established methods for reviews in cardiovascular medicine (PRISMA criteria).

### 2.2. Inclusion and Exclusion Criteria

Studies were included in the current opportunistic review if they met the following criteria: (1) correlating general obesity, VAT, or SAT with the prevalence of carotid artery plaque, (2) evaluating the influence of general obesity, VAT/SAT on carotid artery symptomatology, (3) retrospective or prospective observational clinical studies, (4) performed in humans, (5) full-text available, (6) studies published in English, French, Spanish, and Portuguese.

Other studies were excluded for the following reasons: (1) analyzing intima-media thickness, (2) performed in children, (3) studies reported only as abstracts or with incomplete data, letters, reviews, case reports, nonclinical studies, (4) reviews or meta-analysis.

If the studies had overlapping subjects, the one with the largest sample size was included in the final analysis.

No attempt was made to contact the authors of the included studies to enquire about missing or incomplete data. No studies were excluded because of concerns about missing data.

### 2.3. Data Extraction—Outcomes-Definitions

After removing the duplicated articles, two authors (J.F. and A.C.) independently selected the full-text articles after screening the title and abstract. Disagreements were resolved by consensus. 

For each study, the subsequent data were collected: first author, year of publication, country of the research center, type of study design, total number of patients, age, men (percentages), aims, inclusion and exclusion criteria, and main conclusions (Table 1, Table 2 and Table 3).

Concerning the adipose tissue, the following information was recorded: the method used to determine the overall obesity, the VAT, and SAT (Table 3).

The information collected about carotid stenosis were: imaging methods used to determine the presence of carotid stenosis; the definition of carotid plaque; the grade of carotid stenosis; and if the carotid plaque was symptomatic (Table 3).

The quality of each study was assessed by one author (J. F.) using the MINORS (methodological index for non-randomized studies) criteria (see supplement material) [41]. Each item was scored as 0 (not reported), 1 (reported but inadequate), or 2 (re-ported and adequate). The global ideal score is 16 for non-comparative studies and 24 for comparative studies [41]. The final classification is presented in Table 1.

## 3. Results

**Figure 1:**
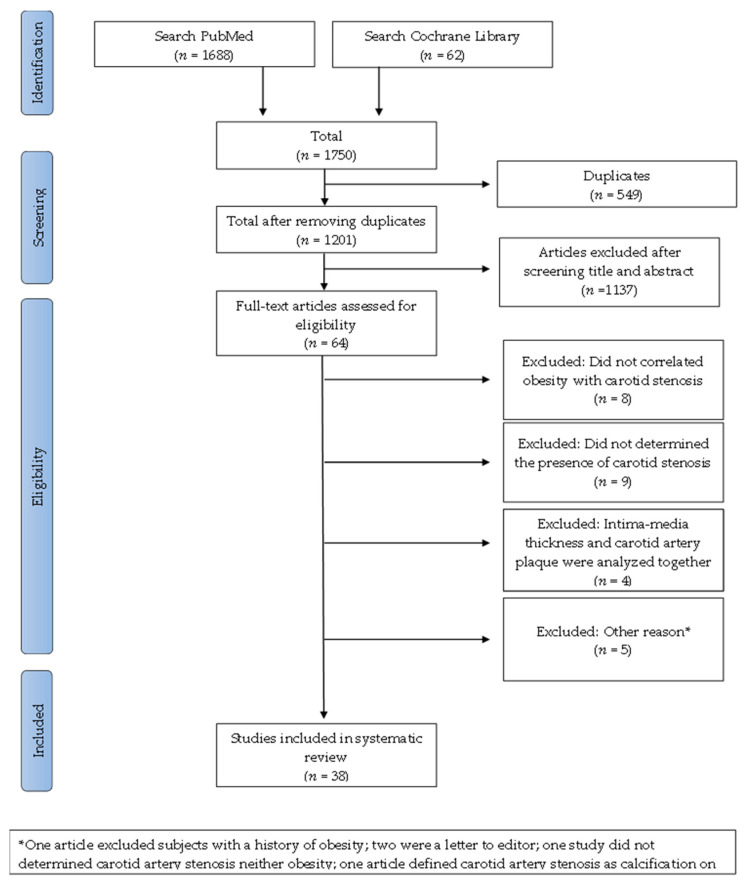
Flow diagram for this opportunistic review that aims to analyze the correlation between obesity and carotid atherosclerosis.

A total of 1750 articles were found, and after removing duplicates, 1201 articles were left, as shown in Figure 1 [42,43,44,45,46,47,48,49].

Of the 64 papers, 26 were rejected after reading the full text due to the following reasons: eight articles did not correlate obesity with carotid artery stenosis [42,43,44,45,46,47,48,49]; Nine did not determine the presence of carotid artery stenosis [50,51,52,53,54,55,56,57,58]; In four articles, intima-media thickness and carotid artery plaque were both correlated with obesity [59,60,61,62]; One article excluded subjects with a history of obesity [63]; Two papers were a letter to the editor [52,64]; One study did not measure carotid artery stenosis or obesity [65]; One article defined carotid artery stenosis as calcification seen on radiographs [66].

The 38 studies included in this opportunistic review were published in 1985 (one article) and between 2001 and 2019. The studies were conducted in 32 different countries, the majority in Europe (18 papers), eight studies in North America, eight in Asia, two in South America, and two in Africa (Table 1).

Analyzing the studies included in this opportunist review by regions, there was a higher number of papers describing a positive association between obesity and carotid plaque characteristics in the research work performed in Asia and Africa. However, just two papers were identified from Africa. The majority of papers included were from Europe, where 10 papers found a relationship and an equal number did not.

- The majority of the studies (34) were cross-sectional, and four were longitudinal.
- A total of 35,339 subjects (49.20% men) were included: 26,492 from Europe, 4239 from Asia, 3859 from North America, 384 from South America, and 365 from Africa (Table 1). Some studies only included a specific group of patients: Type 2 diabetes (5 papers), women (4), patients with an autoimmune disease (4), or hypertension (3) (Table 2).

The authors used different methods to determine the quantity of adipose tissue and to characterize the atherosclerotic plaque. The most frequent method used to determine obesity was anthropometric measures: Thirty-three articles assessed overall obesity with BMI, 19 evaluated visceral obesity with WC, and 11 estimated the relationship between the visceral and subcutaneous adipose tissue with WHR (Table 2). Six authors used medical imaging to quantify the VAT and SAT: Four authors used CT scans [3,8,26,31], one author used ultrasound [40], and another MRI [28]. Other methods were used: bioimpedance (three papers) [9,17,37]; dual-energy X-ray absorptiometry (two) [3,36]; and DXA (one) [8] (Table 2). In one paper, omental, mesenteric, mesocolon, and perirenal fat were dissected after the autopsy and weighed [33]. The VAT was the sum of the omental, mesenteric, mesocolon, and perirenal fat [33].

The carotid plaque was evaluated with ultrasound in 19 articles, with doppler ultrasound in 12 papers, and with MRI in three articles (Table 3).

Two articles analyzed the histological composition of the carotid plaque (number of macrophages, foam cells, cap characteristics, and the quantity of lipids and calcium) [4,19]. One study determined the area of the cadavers of the largest atheroma plaque in the carotid artery of cadavers to calculate the stenosis index [33].

The carotid plaque was defined in most papers as a focal structure encroaching the vessel lumen or as a widening in the intima media-thickness (Table 3). However, there was no homogeneity in the definition, and different publications used different measures of intima-media thickness to define a plaque (Table 3). Sixteen articles only analyzed asymptomatic carotid plaques, six papers studied both symptomatic and asymptomatic, and two articles symptomatic. Fourteen articles did not specify if the carotid artery caused symptomatology. (Table 3). This opportunistic review included 1533 symptomatic and 19,799 asymptomatic patients.

### 3.1. Overall Obesity and the Prevalence of Carotid Plaques

**Figure 2:**
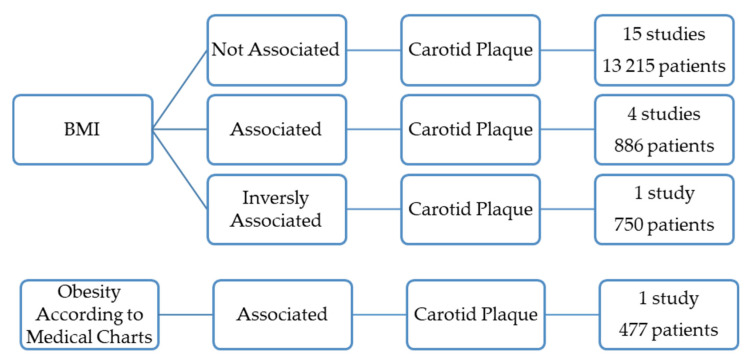
Results of the studies that correlate overall obesity with the presence of the carotid atherosclerotic plaques.

Overall obesity, determined by BMI, was not associated with the presence of carotid plaques in 15 papers, totaling 13,215 patients (Figure 2) [3,9,10,12,14,15,18,21,25,28,30,31,35,39,40].

Five studies (1363 patients) found, however, a positive association between the prevalence of carotid plaques and overall obesity (Figure 2) [5,11,16,27,34].

One of these was a prevalence study conducted on 474 healthy residents in Northeast China [27]. This study sought to determine the risk factors associated with carotid atherosclerosis identified by ultrasound. However, there was no definition of atherosclerotic plaque [27]. The prevalence was significantly higher in obese females than in the control females. Obesity was defined as BMI ≥ 28 kg/m^2^ [27]. The study included 231 males, and no association was found for this gender [27].

Another study conducted on 144 women with systemic lupus erythematous found that the prevalence of carotid artery plaque was significantly associated with obesity, determined by BMI [11].

The oldest study included in this review included 477 patients who performed angiography or Doppler ultrasound and concluded that obesity was significantly more frequent in patients with internal artery occlusion or stenosis than in controls. Obesity was defined according to medical charts [5].

Two papers totaling 268 patients found that the prevalence of carotid plaques was associated with overall obesity determined by BMI [16,34].

However, one paper with 750 individuals concluded that the prevalence of carotid artery plaques was inversely related to BMI (Figure 2) [7].

### 3.2. Overall Obesity and the Characteristics of the Carotid Plaques

**Figure 3:**
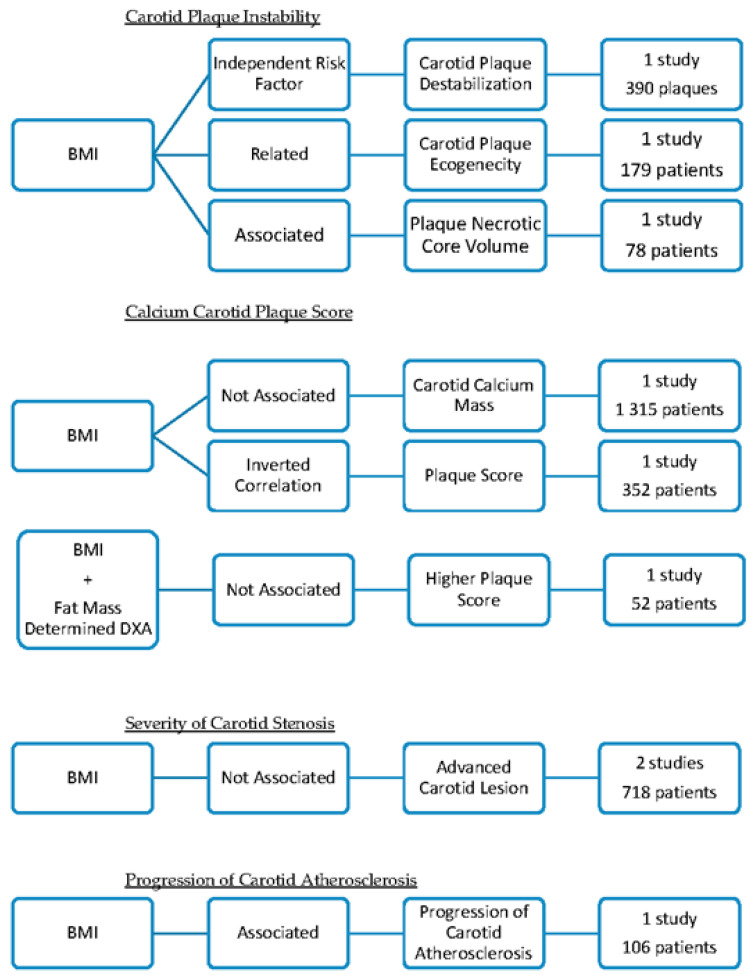
Summary of the studies that correlate overall obesity with the characteristics of the carotid atherosclerotic plaques.

Three papers suggested that overall obesity could be associated with carotid plaque instability (Figure 3) [4,24,36].

(1). Histological analysis of carotid plaques (390) concluded that obesity, defined as BMI ≥ 30 kg/m^2^ was an independent risk factor for carotid plaque destabilization, particularly in males [4]. Obesity was correlated with the presence of unstable carotid plaques, characterized by a high degree of inflammation, thinning, and rupture of the cap [4].
(2). One study analyzed the plaque echogenicity by gray-scale median using ultrasound and concluded that low gray-scale median values were related to high BMI [24]. Plaques with a low gray-scale median had a higher probability of causing embolization and symptoms. This research included 179 diabetic patients [24].
(3). One research paper found that obesity (BMI > 30.0 kg/m^2^) was associated with increased carotid plaque necrotic core volume and calcification independently of diabetes mellitus status [36]. The carotid plaque composition was assessed by magnetic resonance imaging. Obesity was determined by BMI [36]. The study included 78 patients with short-duration Type 2 diabetes mellitus and 91 sex- and aged-matched control subjects [36].

This opportunistic review identified three articles that found that obesity did not correlate with calcium score or carotid plaque score (Figure 3) [8,26,37].

(1). No association was found between BMI and calcium mass score of the carotid arteries determined with a CT scan [26]. This study was performed on 1315 diabetic patients [26].
(2). One study found that the measures of adiposity (BMI, the systemic fat mass, and the fat-free mass determined with DXA) were not significantly different in patients with higher plaques score [8]. For each segment, the degree of plaque was graded as follows: 0 = no plaque; 1 = 1 small plaque. <30% of vessel diameter; 2 = 1 medium plaque between 30% of vessel diameter or multiple small plaques; and 3 = 1 large plaque >50% of the vessel diameter or multiple plaques with at least 1 medium plaque [8]. The grades were summed across the right and left carotid arteries to create an overall measure of the extent of focal plaque [8]. This study included 52 patients [8].
(3). One paper found an inverse correlation between BMI and plaque score, defined as the total plaque thickness for the visualization sites in the IMT measurement on the right and left size [37]. The study was performed on 352 rheumatoid arthritis patients [37].

Two different papers did not find any relation between BMI and the severity of atherosclerotic plaque (Figure 3) [19,23].

(1). A histological study analyzed the relationship between obesity and the severity of atherosclerosis carotid plaque [19]. The atherosclerotic lesions were described according to the American Heart Association classification [19]. BMI did not independently predict the risk of developing advanced carotid atherosclerotic lesions (including 185 cadavers of men and women) [19].
(2). No association was found between obesity (BMI > 27 kg/m^2^) and carotid stenosis ≥ 50% (determined with NASCET criteria) in 533 patients [23]. In this study, there was no association between obesity and symptomatic carotid stenosis [23].

Another paper concluded that obesity (determined by BMI) was associated with the progression of carotid atherosclerosis, studied with magnetic resonance [29]. This research project determined the change in the carotid artery wall in 106 hyperlipidemic participants during the course of treatment with statins (Figure 3) [29].

### 3.3. Visceral Adipose Tissue and the Prevalence of Carotid Plaque

Nineteen articles analyzed the relationship between VAT and carotid stenosis.

**Figure 4:**
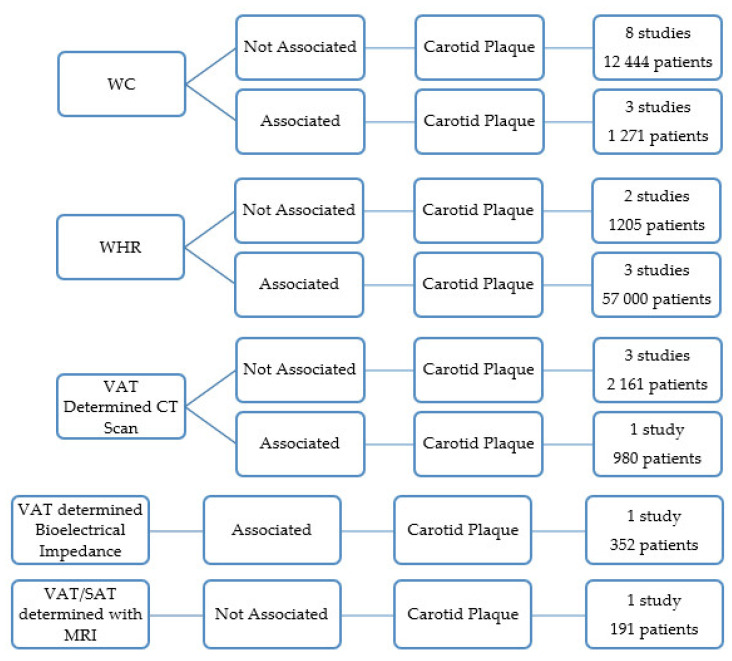
Results of the papers correlating visceral obesity with the presence of the carotid atherosclerotic plaques.

Eight papers performed in 12,444 patients showed no association between waist circumference (WC) and the presence of carotid plaques [9,13,15,16,22,25,35,39,40]. Three studies, however, found that the presence of plaque was associated with WC in 1271 subjects (Figure 4) [7,21].

Three other studies that included 5 700 subjects found that WHR was associated with the prevalence of carotid plaques (Figure 4) [15,35,38].

WHR was significantly related to the presence of carotid artery plaque in African Caucasian women, whereas none of the obesity measures were associated with carotid artery plaque in black women [18]. The study was limited to 203 African black and African Caucasian women who met the American College of Rheumatology criteria for rheumatoid arthritis (Figure 4) [18].

In two research works, there was no association between WHR and the presence of carotid plaques (1205 subjects) (Figure 4) [9,28].

A research work analyzed the relationship between new anthropometric measures that reflected the quantity of abdominal adipose tissue and the carotid plaque [39]. The anthropometric measures were the A Body Shape Index (ABSI) and Body Roundness Index (BRI) [39]. ABSI was independently associated with the presence of carotid atherosclerotic plaque. The study included 468 subjects with arterial hypertension [39].

Four articles used CT scans to quantify the VAT and another used MRI [3,8,26,28,31]. The VAT area determined with a CT scan in 980 healthy Japanese was independently associated with cervical plaque [31]. In three studies with 2161 subjects, the VAT area determined with a CT scan was not associated with the presence of carotid plaques [3,8,26]. One paper published the relationship between VAT and SAT as determined by MRI and the presence of carotid plaque [28]. The study included 191 subjects, and no relation was found between the variables (Figure 4) [28].

One paper concluded that VAT measured with bioelectrical impedance was associated with a higher prevalence of atherosclerotic carotid plaques [37]. This study was limited to 352 patients with rheumatoid arthritis (Figure 4) [37].

### 3.4. Visceral Adipose Tissue and the Characteristics of the Carotid Plaque

**Figure 5:**
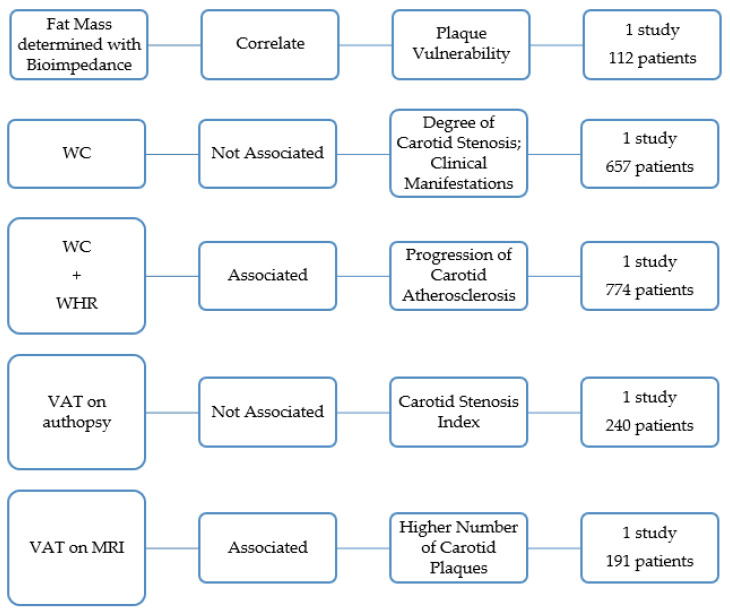
Overview of the studies that analyze the association between visceral obesity and the characteristics of the carotid atherosclerotic plaques.

A research work of 112 subjects found that increased fat mass (determined with bioimpedance) correlates with carotid plaque vulnerability, as expressed by the gray scale median score (Figure 5) [17].

A research work determined WC in 657 patients with symptoms of cerebral ischemia and carotid stenosis of ≥50% [20]. The author concluded that patients with and without abdominal obesity did not significantly differ either in the degree of carotid stenosis or in the degree of its clinical manifestation [20]. Haberka also reached a similar conclusion determining the central obesity with WC and ultrasound [8]. In this study, the amount of visceral fat was not related to carotid stenosis severity (Figure 5) [40].

A study in 774 men without atherosclerosis showed that abdominal obesity, as indicated by high WHR and high WC, was associated with accelerated progression of carotid atherosclerosis independent of overall obesity and other risk factors in middle-aged men with no prior atherosclerotic diseases (Figure 5) [6].

An autopsy study with 240 deceased subjects concluded that VAT was not associated with carotid artery stenosis index [33]. The VAT was the sum of the weight of the omental, mesenteric, mesocolon and perirenal fat [33]. The largest atheroma plaque in the carotid artery was determined [33]. The stenosis index was calculated by subtracting the lumen area from the outer area, dividing the difference by the outer area, and multiplying the result by 100 (Figure 5) [33].

One paper showed that subjects with the highest amount of VAT were more prone to have more than one carotid plaque compared to participants showing the highest values of SAT or other conventional anthropometric indices [28]. The quantity of VAT and SAT was quantified by MRI in 191 subjects (Figure 5) [28].

### 3.5. Subcutaneous Adipose Tissue and Carotid Plaque

Six studies evaluated SAT, and none found any relationship with carotid plaques [3,8,26,28,31,40].

## 4. Discussion and Conclusions

To the best of our knowledge, this is the first opportunistic review analyzing the relationship between obesity and carotid artery disease.

The general assumption emerging from our analysis suggests no association between overall obesity (determined with BMI) or visceral obesity (determined in the majority of the studies with WC) and the presence of carotid plaque. However, three studies (5700 patients) found that WHR was associated with the prevalence of carotid plaques [15,35,38]. BMI is not always a measure of fatness. Individuals with more muscle mass may be incorrectly classified as obese [67]. BMI has poor specificity for excess adiposity, and it does not characterize the excess of centrally distributed obesity [68]. Low BMI values may also be associated with the loss of lean body mass (muscle) [68]. In contrast with BMI, WC and WHR specifically address abdominal obesity and correlate better with overall atherosclerotic disease prevalence [68]. WC and WHR may correlate better with body fatness [67] and may more accurately reflect the additional risk conferred by obesity [68]; actually, they are more associated with mortality and cardiovascular events than BMI [68].

Although WC is well-described as a measure of VAT and a marker of obesity’s associated metabolic risks, WHR has superior performance in estimating atherosclerotic risk [68]. One possible explanation is that WHR is an indexed value (to lower body girth) and provides a more precise assessment of relative central adiposity across the spectrum of body size, compared with WC [68]. WHR is considered the main anthropometric measure of central obesity [15,35,38]. Another explanation is that increased hip circumference may protect against atherosclerosis [68]. Fat in the lower body may function as a protective reservoir against ectopic (abdominal) adiposity [68]. WHR is independently associated with prevalent atherosclerosis and provides better dis- crimination than either BMI or WC [68].

There is a correlation between the WHR and the ratio of VAT-to-SAT cross-sectional area (quantified by CT images taken in the abdominal region) [69]. VAT is an endocrine organ that can secrete adipokines, including cytokines and chemokines [70,71]. VAT is associated with cardiovascular disease and can be used as a cardiometabolic risk marker, while SAT has a beneficial metabolic impact in the opposite direction [71]. Abdominal visceral fat correlates with the prevalence of coronary artery disease and mortality. Subcutaneous fat may play a protective role against the development of coronary artery disease by improving insulin sensitivity or the secretion of adipokines. The VAT/SAT ratio is a unique parameter relevant to vascular inflammation or poor cardiovascular outcomes [71,72]. VAT/SAT ratio is associated with a higher total mortality and incidence of MACE, independently of traditional vascular risk factors, and with the presence of obstructive coronary artery disease [2].

In this review, we found a relationship between obesity and other characteristics of carotid atherosclerotic plaque. Overall obesity (738 patients) [4,24,36] and an increase in fat mass (112 patients) [17] were associated with carotid plaque instability. Obesity and visceral fat (880 patients) are correlated with the progression of carotid atherosclerosis [6,29]. However, no study related obesity to carotid artery symptomatology.

Studies conducted with cardiac patients found that obesity was also positively associated with the progression of coronary plaque [72,73]. Excess VAT is correlated with higher serum levels of fasting glucose, triglycerides, lower HDL cholesterol, greater prevalence of hypertension, tobacco use, and artery inflammation [72,73]. The systemic inflammation causes endothelial dysfunction, formation, and progression of atherosclerotic plaques [73,74]. Importantly, inflammation is also responsible for atherosclerotic plaque instability [43]. These biological facts could also explain why in this opportunistic review, we found that obesity did not correlate with calcium score, carotid plaque score [8,26,37], or the severity of atherosclerotic plaque [19,20,23,40].

None of the studies included in this review found any relationship between SAT and carotid plaques [3,8,26,28,31,40]. Further studies to characterize the subcutaneous fat should be developed.

### 4.1. Strengths and Limitations

A key strength of this review is the standardized data extraction, the quality assessment procedures, and searches conducted by two authors. This manuscript covers a broader range of patients and countries from 1985 to 2019 (most articles were published in the last 10 years). It includes published research in English, French, Portuguese, and Spanish language journals. The main limitation of this revision is that it did not conduct a meta-analysis. The articles included are not homogenous. There are different definitions of carotid plaque and obesity. The authors included different subjects and used different methods to determine obesity and carotid plaque. The opportunistic review included observational studies, and its bias was not entirely avoided.

Another bias of this study is the inability to determine the impact of obesity on atherosclerotic plaque independently of the cardiovascular risk. Obesity can increase the prevalence of certain factors such as dyslipidemia which contributes to atherosclerosis. However, there was no description of cardiovascular risk factors in all included papers.

Another limitation is that two research questions were not answered: (i). We did not find the relation between VAT and symptomatic carotid artery disease (ii). neither was the role played by subcutaneous tissue in carotid stenosis.

### 4.2. Implications for Practice

The results of the current investigation provide key information for practice. First, the importance of determining the WHR to infer the relation between VAT and SAT. The BMI, more frequently used in clinical activity, can erroneously represent the cardiovascular risk. Secondly, it may be useful to address obese patients to increase lean body mass. This study also has an impact on future research. The authors should use rigorous methods to determine obesity to homogenize the results, and facilitate the comparisons accurately. The role of subcutaneous tissue in carotid atherosclerosis still needs more investigations to clearly determine its role in vascular diseases. The relationship between obesity, subcutaneous fat, and carotid symptomatology should be more deeply investigated. Behavioral and pharmacological interventions could be developed to decrease the VAT/SAT ratio. Studies focused on obesity and inflammation could be important in atherosclerosis control.

### 4.3. Concluding Remarks

Considering the data analyzed, obesity and visceral obesity were not associated with the presence of carotid plaque. The ratio between VAT and SAT could influence the prevalence of carotid plaques. Obesity could be related to carotid plaque instability and progression, but its association with carotid symptomatology has not been proved and should be investigated in future studies.

## Figures and Tables

**Table 1:** Characteristics of the studies included in the opportunistic review.

Study	Year	Country	Type of Study	Total Number of Patients	Quality of Studies MINOR CRITERIA
Bogousslavsky et al. [5]	1985	Switzerland	Cross-sectional	477	3
Lakka et al. [6]	2001	Finland	Longitudinal	774	12
Hunt et al. [7]	2002	USA	Cross-sectional	750	14
Hegazi et al. [8]	2003	USA	Cross-sectional	52	12
Czernichow et al. [9]	2005	France	Cross-sectional	1014	13
Hadjiev et al. [10]	2003	Bulgaria	Cross-sectional	500	14
De Souza et al. [11]	2005	Brazil	Cross-sectional	144	14
Montalcini et al. [12]	2006	Italy	Cross-sectional	313	14
Lear et al. [12]	2007	Canada	Cross-sectional	794	14
Park et al. [13]	2007	Korea	Cross-sectional	378	14
Irace et al. [14]	2009	Italy	Cross-sectional	1842	12
Yu et al. [15]	2009	Hong Kong	Cross-sectional	518	13
Terzis et al. [16]	2011	Greece	Longitudinal	106	14
Kadoglou et al. [17]	2012	Greece	Longitudinal	112	12
Solomon et al. [18]	2012	South Africa	Cross-sectional	203	12
Rodríguez-Flores et al. [19]	2013	Mexico	Cross-sectional	185	14
Maksimovic et al. [20]	2013	Serbia	Cross-sectional	657	14
Galarza-Delgado et al. [21]	2013	Mexico	Cross-sectional	124	14
Cuspidi et al. [22]	2013	Italy	Cross-sectional	3752	12
Chiquete et al. [23]	2014	Mexico	Cross-sectional	533	12
Irie et al. [24]	2014	Japan	Cross-sectional	179	12
Yan et al. [25]	2014	China	Cross-sectional	911	12
Yuan et al. [26]	2016	USA	Cross-sectional	1315	12
Pan et al. [27]	2016	China	Cross-sectional	474	12
Radmard et al. [28]	2016	Iran	Cross-sectional	191	12
Sandfort et al. [29]	2016	USA	Longitudinal	106	12
Mitevska et al. [30]	2017	Macedonia	Cross sectional	60	14
Higuchi et al. [31]	2017	Japan	Cross-sectional	980	12
Mancusi et al. [32]	2017	Italy	Cross-sectional	8815	14
Nishizawa et al. [33]	2017	Brazil	Cross-sectional	240	12
Omisore et al. [34]	2018	Nigeria	Cross-sectional	162	14
Imahori et al. [35]	2018	Norway	Cross-sectional	4906	12
Laugesen et al. [36]	2018	Denmark	Cross-sectional	169	12
Yoshida et al. [37]	2018	Japan	Cross-sectional	352	14
Scicali et al. [38]	2018	Italy	Cross-sectional	276	12
Rovella et al. [4]	2018	Italy	Cross-sectional	390	12
Geraci et al. [39]	2019	Italy	Cross-sectional	468	14
Haberka et al. [40]	2019	Poland	Cross-sectional	391	14

**Table 2:** Study aims, inclusion and exclusion criteria, methods to study adipose tissue.

Study	Age (Years)	Men (%)	Aims	Criteria		Method Used to Determine		
				Inclusion	Exclusion	Overall Obesity	VAT	SAT
Bogousslavsky et al. [5]	62.1	363 (76.1%)	Determine the relative importance of each cardiovascular risk factor according to the progression of local atheromatous obstruction	Patients with atheromatous internal carotid artery occlusion or stenosis, compared with matched control subjects without internal carotid artery disease and with matched patients with coronary heart disease but without internal carotid artery disease	Age < 50 years old, without anatomic verification of stenosis, with dissection, dysplasia, posttraumatic occlusion, or intake of oral contraceptives	According to medical charts	NA	NA
Lakka et al. [6]	NA	774 (100%)	Whether WHR and WC are directly related to a 4-year increase in the indicators of common carotid atherosclerosis independent of BMI and other risk factors for atherosclerosis	Men, 42–60 years old, Complete information about anthropometric measures and carotid atherosclerosis	Coronary heart disease, stroke, claudication	BMI	WC WHR	HC WHR
Hunt et al. [7]	42.2 ± 15.9	289 (38.5%)	The extent to which the presence or absence of carotid artery plaque was under genetic control	Age: 40–60-year-old, that the proband has a living spouse who was willing to participate in the study, and that the proband has at least 6 first-degree relatives, excluding parents, who were at least 16 years of age and living in the San Antonio area	NA	BMI	WC	NA
Hegazi et al. [8]	51 ± 9	18 (35%)	Examine the relationship between obesity and regional patterns of adiposity, insulin resistance, and five independent measures of subclinical atherosclerosis	Volunteers with a prior diagnosis of Type 2 diabetes of known duration not longer than 5 years, age: 20–70 years, and stable weight, with overall good general health	Insulin treatment, current use of tobacco, prior history of myocardial infarction, stroke, or peripheral vascular disease	BMI DXA	CT scan.	CT scan.
Czernichow et al. [9]	59.4 ± 4.7	504 (49.7%)	Association of body composition assessed by bioimpedance analysis and anthropometric indicators of fat repartition with carotid structure and function	Volunteers Women aged: 35–60 years Men aged: 35–60 years	Disease likely to hinder participation or threaten 5 years survival. Extreme beliefs or behavior regarding diet	Bioimpedance BMI	WC WHR	HC WHR
Hadjiev et al. [10]	2003	NA	This population-based biennial epidemiological survey has been designed to assess the prevalence of the multiple vascular risk factors, their distribution patterns, and outcomes among the Bulgarian urban population.	Without signs and symptoms of cerebrovascular disease, aged 50–79 years were enrolled in the study	NA	BMI	NA	NA
De Souza et al. [11]	34.0 ± 11.7	0 (0%)	Estimated the prevalence of atherosclerotic plaque in carotid arteries in systemic lupus erythematous patients and controls and verified possible associations between risk factors and carotid plaque	Women fulfilled the update American College of Rheumatology criteria for systemic lupus erythematosus	Controls excluded if they had an autoimmune disease	BMI	NA	NA
Montalcini et al. [12]	57.2 ± 7.37	0 (0%)	Investigate whether the subclinical carotid atherosclerosis prevalence is different in obese postmenopausal women with and without metabolic syndrome	Postmenopausal, Caucasian, aged 45–75 years	Diabetes cardiovascular disease arrhythmia	BMI	NA	NA
Lear et al. [3]	46.9 ± 8.7	389 (48.6%)	Hypothesized that the association between VAT and atherosclerosis is independent of total body fat, established risk factors, and measures of central adiposity	Healthy men and women (between 30 and 65 years of age) matched for ethnicity and BMI	Recent weight change, previous diagnosis of cardiovascular disease, significant comorbidity, had significant prosthetics or amputations, currently taking medications for cardiovascular risk factors	Dual-energy X-ray absorptiometry, CT scan BMI	CT scan WC WHR	CT scan WHR
Park et al. [13]	65.3 ± 12.2	204 (54%)	Elucidate the relationship between metabolic syndrome and cerebrovascular stenosis	Consecutive patients with acute ischemic stroke (large artery atherosclerosis, small artery occlusion, cardiac embolism, and ischemic stroke of undetermined etiology)	Strokes of other determined etiology (venous thrombosis, arterial dissection, or moyamoya disease and those with transient ischemic attack). Patients unable to stand with assistance, patients who had no relevant lesions on diffusion-weighted imaging, poor MR angiographic images, incomplete work up	NA	WC	NA
Irace et al. [14]	30–80	1002 (54.4%)	Evaluate the contribution of generalized adiposity, to carotid atherosclerosis, in participants with or without metabolic syndrome	Caucasians	BMI < 18.5 kg/m^2^_,_ age < 30 years	BMI	WC	NA
Yu et al. [15]	56.4 ± 3.3	0 (0%)	Determine the prevalence of carotid plaque and identity its associated risk factors	Postmenopausal Chinese women aged 50–64 years	Surgical menopause, presence of cardiovascular disease, cancer and renal failure	BMI	WC WHR	WHR
Terzis et al. [16]	40.5 ± 1.1	60 (56.6%)	Assess associations between actual long-term changes in BMI since adolescence and early and advanced stages of subclinical atherosclerosis among a population of healthy young adults	This longitudinal study was based on a cohort initially recruited consecutively from two Athens high schools in 1983, collecting data on cardiovascular risk factors from a population of consecutive adolescents aged 12–17 years	Loss/change of contact information or decline to participate, or not alive	BMI	WC	NA
Kadoglou et al. [17]	65 ± 7.7	86 (76.8%)	Assess if apelin and visfatin correlate with carotid plaque echogenicity	Aged 56–80 years and overweight (BMI > 25 kg⁄m^2^ fat-mass > 30%) and with unilateral or bilateral carotid atherosclerosis without indications for intervention, not receiving lipid-lowering treatment	Cerebral hemorrhage, sources of cardioembolism, concurrent conditions, diseases interfering with the expression of inflammatory mediators during the previous 3 months	BMI Bioimpedance	WHR	WHR
Solomon et al. [18]	56.4 ± 10.9	0 (0%)	Ascertain the association between clinical obesity and atherosclerosis	African black women and Caucasian women who met the American College of Rheumatology criteria for rheumatoid arthritis	Infected with HIV	BMI	WC WHtR WHR	WHR
Rodríguez-Flores et al. [19]	NA	107 (57.8%)	Analyze the relationship between cardiovascular risk factors, including obesity, with the severity of atherosclerosis in different arterial territories	Cadavers of men and women aged 0 to 90 years	Arteries were not taken for examination in the following circumstances: when tissues had suffered advanced damage that precluded their analysis when the cadaver arrived more than 36 h after death, or in cases with previously reported congenital heart disease	BMI	NA	NA
Maksimovic et al. [20]	65.3 ± 8.4	412 (62.7%)	Investigate the relationship between abdominal obesity, and other atherosclerotic risk factors in patients with symptomatic carotid atherosclerotic disease	Subjects who had symptoms of cerebral ischemia and carotid stenosis of ≥50%	Age < 18 years, malignant disease, rheumatoid arthritis, or previous endarterectomy	NA	WC	NA
Galarza-Delgado et al. [21]	55.5 ± 13.1	13 (10.5%)	Association between the presence of rheumatoid nodules and plaque of the carotid artery	Met at least 4 American Colege of Rheumatology criteria for rheumatoid arthritis greater than 16 years	Pregnant patient, History of carotid surgery	BMI	WC	NA
Cuspidi et al. [22]	53.3 ± 12.6	1977 (52.7%)	Risk of developing left ventricular hypertrophy and carotid atherosclerosis is different in men and women with metabolic syndrome	Uncomplicated essential hypertension	Previous clinically overt cardiovascular disease, secondary causes of hypertension, life-threatening conditions	NA	WC	NA
Chiquete et al. [23]	69.2	211 (39.6%)	Identify risk factors associated with moderate to severe carotid stenosis	History of ischemic stroke or transient ischemic attack or at least two cardiovascular risk factors (Age ≥ 55 years, hypertension, dyslipidemia, smoking habits, obesity or diabetes)	NA	BMI	NA	NA
Irie et al. [24]	65 ± 7	147 (82%)	Clarify the parameters related to the echogenicity of carotid plaque	Age ≥ 40 years, Type 2 diabetes, presence of carotid plaques	History of ischemic stroke, coronary heart disease, peripheral artery disease, elevated liver enzymes, renal insufficiency	BMI	NA	NA
Yan et al. [25]	68.1 (4.9)	370 (40.6%)	Investigate the association of the metabolic syndrome components with subclinical atherosclerosis	Age ≥ 60 years	Patients with clinical stroke, coronary heart disease, or heart failure	BMI	WC	NA
Yuan et al. [26]	58.9 (9.7)	552 (42.0%)	Assess relationships between anthropometric measures and adipose tissue volumes with subclinical cardiovascular disease in carotid arteries	Type 2 diabetes	Prior coronary artery procedures. Absence of coronary artery calcification	BMI	WC CT scan	CT scan
Pan et al. [27]	NA	231 (48.7%)	Identify risk factors associated with carotid atherosclerosis	Relatively healthy populations residing in Northeast China	Excessive alcohol consumption, severe hepatitis B or C, liver disease, mental illness, severe cardiac or pulmonary insufficiency, and cancer	BMI	WC	NA
Radmard et al. [28]	57 ± 5.7	92 (48.2%)	Association between quantitative measures of central adiposity with indicators of carotid atherosclerosis	Aged over 50	Contraindications for MRI	BMI	WHR WHtR MRI	WHR WHtR MRI
Sandfort et al. [29]	65	67 (63%)	Evaluate the change of atherosclerosis in the carotid artery wall in hyperlipidemic participants during treatment with statins and determine cardiovascular risk factors associated with change in extent of atherosclerosis	Age ≥ 55 years and an indication for lipid-lowering therapy	Contraindication for statin therapy, use of nonstatin lipid-lowering therapy, and ineligibility for MRI scan	BMI	NA	NA
Mitevska et al. [30]	67 ± 6	34 (56.7%)	Evaluate the risk factor profile, presence of asymptomatic carotid artery disease, and predictors of coronary artery disease in asymptomatic Type 2 diabetic patients	Asymptomatic patients with Type 2 diabetes	Typical stable angina pectoris, previously known or established as coronary artery disease, left ventricular ejection fraction < 50% at rest, severe valvular disease, atrial fibrillation, left bundle branch block, presence of a pacemaker, severe chronic pulmonary disease	BMI	NA	NA
Higuchi et al. [31]	59.0 ± 11.5	655 (67%)	Evaluate the clinical impact of visceral fat accumulation on the cerebrovascular lesions	Japanese aged ≥ 40 years	NA	BMI	WHR CT scan	WHR CT scan
Mancusi et al. [32]	54.0 ± 11.5	5104 (57.9%)	Impact of obesity on carotid target organ damage	Hypertensive patients without prevalent cardiovascular disease	Cardiovascular disease: myocardial infarction, angina, coronary revascularization, stroke, transitory ischemic attack, or congestive heart failure requiring hospitalization	BMI	NA	NA
Nishizawa et al. [33]	64.8 ± 15.3	151 (62.9%)	Investigate the association between abdominal visceral fat with atherosclerosis in the aorta, coronary, carotid, and cerebral arteries in an autopsy study	Aged ≥ 30 years	Family provided inconsistent information during the clinical interview, the family had less than weekly contact with the deceased, the next of kin was unable to participate due to emotional suffering, subjects who had lost 10% or more of regular weight during the six months prior to death, arteries or visceral fat was retained at autopsy by the pathologist, subjects with post mortem interval ≥ 24 h, and subjects with signs of body autolysis according to the Crossley criteria	NA	Omental, mesenteric, mesocolon, and perirenal fat were dissected after the autopsy and weighed. The VAT was the sum of the omental, mesenteric, mesocolon and perirenal fat	NA
Omisore et al [34].	52.0 ± 15.1	80 (49.4%)	Evaluated the impact of traditional cardiovascular risk factors on carotid atherosclerosis in a sample of Nigerian adults	Adults aged 18 years and older	NA	BMI	NA	NA
Imahori et al. [35]	66 (60–72)	2184 (44.5%)	Evaluate the associations between adiposity measures and the presence of carotid plaque	Right carotid artery	NA	BMI	WC, WHR, WHtR	WHR, WHtR
Laugesen et al. [36]	59 ± 9.4	86 (50.9%)	Assess plaque composition by carotid magnetic resonance imaging	Age > 18 years, diagnosis of Type 2 diabetes mellitus and known duration of diabetes mellitus < 5 years	Acute or chronic infectious disease, end-stage renal failure, pregnancy or lactation, prior or present cancer, and contraindications to MRI (including body weight > 120 kg).	Fat percentage was assessed by whole-body dual-energy X-ray absorptiometry. BMI	WHR	WHR
Yoshida et al. [37]	61.8 ± 11.9	60 (17%)	Determine the association between obesity and/or VAT and the risk for atherosclerosis	Age > 18 years, Japanese rheumatoid arthritis patients	Presence of an internal or external electronic device, severely degraded health status, presence of fractures and pain preventing assessment of visceral and subcutaneous fat, WC < 57 cm, poor physical health on the day of examination, and concurrent cancer and hepatitis treatment, dialysis, and/or sex-hormone suppression or replacement therapy	BMI	Bioimpedance	Bioimpedance
Scicali et al. [38]	56.8 ± 8.0	183 (66.3%)	Investigate the presence of carotid plaque in overweight patients	BMI: 25–29.9 kg/m^2^, age: 40–70 years, and at least one cardiovascular risk factor (hypertension, dyslipidemia, or current smoking)	Previous history of diabetes, coronary heart disease, cerebrovascular disease, peripheral artery disease, or clinical evidence of advanced renal disease	BMI	WHR WC	NA
Rovella et al. [4]	69.8 (7.2)	273 (70%)	Evaluate by histology the role of obesity in the destabilization of carotid plaques	Submitted to carotid endarterectomy AND Symptomatic patients with thrombo-embolism due to carotid atherosclerosis OR Asymptomatic patients with carotid stenosis ≥ 60%	Cardiac source of embolization, stenosis greater than 50% of Willis circle	BMI	NA	NA
Geraci et al. [39]	58 ± 14	279 (59.6%)	Relationship between anthropometric indices of adiposity and carotid atherosclerosis	Caucasian patients, age: 30–80 years with essential hypertension	BMI > 40 kg/m^2^, Renovascular, endocrine, or malignant hypertension, Carotid thromboendarterectomy and/or percutaneous carotid angioplasty, Pre-existing cardiovascular comorbidities	BMI BSA	ABSI BRI WC	WHtR
Haberka et al. [40]	61.8 ± 8	255 (65.2%)	Association between obesity, fat depots, and carotid artery stenosis in patients with high cardiovascular risk	Scheduled for elective coronary angiography	Heart failure, severe primary heart valve disease or any other extracardiac chronic disease causing at least 10% unintentional weight loss (prior 3 months), secondary causes of obesity or medical intervention aimed at weight loss, neck or abdomen surgery, neck radiotherapy, a very poor carotid artery image quality, and confirmed diagnosis of a genetic predisposition for CV diseases	BMI	Ultrasound WC	Ultrasound

**Table 3:** Data collected about carotid plaque and conclusion.

Study	Carotid Plaque				Conclusion
	Evaluation Method	Definition	Grade	Symptomatic	
Bogousslavsky et al. [5]	Angiography US doppler	Defined on radiological clinic grounds and confirmed pathologically in patients who underwent endarterectomy	Determined	Symptomatic (157 patients) Asymptomatic (320 patients)	Obesity was significantly more frequent in patients with internal artery occlusion or stenosis than in controls
Lakka et al. [6]	Ultrasound	Plaque height was calculated as the average of the differences between the maximal and minimal IMT of the right and left common carotid artery and was used as an indicator of how steeply atherosclerotic lesions protruded into the lumen	NA	Asymptomatic	Abdominal obesity, as indicated by high WHR and by high WC, is associated with accelerated progression of carotid atherosclerosis independent of overall obesity and other risk factors in middle-aged men with no prior atherosclerotic diseases
Hunt et al. [7]	US doppler	Focal widening of the IMT relative to the adjacent wall segment, measuring at least 1.5 mm in thickness	NA	Symptomatic and asymptomatic	The prevalence of carotid artery plaque increased with WC and decreased with BMI
Hegazi et al. [8]	Ultrasound	Plaque was defined as a distinct area of hyperechogenicity and/or protrusion into the lumen of the vessel with at least 50% greater thickness than the surrounding area	Plaque was defined as a distinct area of hyperechogenicity and/or protrusion into the lumen of the vessel with at least 50% greater thickness than the surrounding. For each segment, the degree of plaque was graded as follows: 0 = no plaque, 1 = 1 small plaque. <30% of vessel diameter, 2 = 1 medium plaque between 30% of vessel diameter or multiple small plaques, and 3 = 1 large plaque >50% of the vessel diameter or multiple plaques with at least 1 medium plaque. The grades were summed across the right and left carotid arteries to create an overall measure of the extent of focal plaque	Asymptomatic	Measures of adiposity were not significantly different in patients with higher plaques score
Czernichow et al. [9]	Ultrasound	Localized eco structures encroaching upon the vessel lumen for which the distance between the media-adventitia internal side of lesion was >1 mm	NA	NA	No association was found between the presence of carotid plaques and body composition
Hadjiev et al. [10]	Duplex scanning was employed	NA	Classification of carotid stenosis according to NASCET	Asymptomatic	Obesity was not associated with asymptomatic carotid stenosis of 50% or greater
De Souza et al. [11]	Ultrasound	Distinct area of hyperechogenicity and/or a focal protrusion of the vessel wall into the lumen	NA	Symptomatic (11 patients) Asymptomatic (133 patients)	The prevalence of carotid artery plaque was significantly associated with obesity
Montalcini et al. [12]	US doppler	Was defined as an echogenic focal structure encroaching the vessel lumen with a distinct area 50% greater than the intima-media thickness of neighboring sites. Stenosis was defined as a peak systolic velocity >120 cm/s, and occlusion was defined as the absence of a Doppler signal	NA	Asymptomatic	BMI was not associated with carotid atherosclerosis Women with metabolic syndrome who were overweight or obese had approximately three times higher adjusted odds of having carotid atherosclerosis
Lear et al. [3]	Ultrasound	Focal plaques were identified as wall thickness that was increased compared with the surrounding IMT	NA	Asymptomatic	There were no differences in BMI, total fat mass, percent body fat, total abdominal adipose tissue, and SAT in those with versus without carotid plaques. In those with plaques, VAT was a significant, independent predictor of plaque area after adjusting for age, sex, ethnicity, education, household income, family history of CVD, smoking, and percent body fat
Park et al. [13]	Magnetic resonance	Degree of luminal narrowing of ≥ 50%	NA	Symptomatic	None of the metabolic syndrome components were shown to be associated with extracranial internal carotid artery stenosis
Irace et al. [14]	US doppler	Localized lesion encroaching the lumen of thickness at least 1.3 mm, no spectral broadening or only in the deceleration phase of systole and systolic peak velocity less than 120 cm/s. Stenosis was defined as spectral broadening throughout systole and/or peak flow velocity of at least 120 cm/s	NA	NA	Overweight and obesity, however, do not independently associate with carotid atherosclerosis
Yu et al. [15]	Ultrasound	Plaque was defined as a focal wall thickening of at least 1.5 mm	The degree of plaque at the six segments was graded according to the following criteria: grade 0, no observable plaque, grade 1, one small plaque < 30% of vessel diameter, grade 2, one medium plaque between 30% and 50% of the vessel diameter or multiple small number plaques, and grade 3, one large plaque > 50% vessel diameter or multiple plaques with at least one medium plaque	Asymptomatic	A high WHR was independently associated with the presence of plaque
Terzis et al. [16]	Ultrasound	Plaque was defined as a focal structure encroaching into the arterial lumen of at least 0.5 mm or 50% of the surrounding IMT value, or a thickness of 1.5 mm as measured from the media-adventitia interface to the intima-lumen interface	NA	Asymptomatic	The presence of atheromatous plaques was independently associated with BMI
Kadoglou et al. [17]	US doppler	Localized thickening of the vessel wall of more than 2.5 mm	Classification of carotid stenosis according to the recommendations of the Society of Radiologists in Ultrasound	Symptomatic (35 patients) Asymptomatic (51 patients)	Increased fat mass correlated with carotid plaque vulnerability, as expressed by the gray scale median score
Solomon et al. [18]	Ultrasound	Focal structure that encroaches into the arterial lumen of a least 0.5 mm or 50% of the surrounding intima-media thickness value or demonstrates a thickness of >1.5 mm as measured from the media-adventitia interface to the intima-lumen interface	NA	NA	WHR was significantly related to carotid artery plaque in African Caucasian women, whereas none of the obesity measures were associated with carotid artery plaque in black women
Rodríguez-Flores et al. [19]	Histopathological study	Classification of atherosclerosis lesions according to the American Heart Association	NA	NA	BMI did not independently predict the risk of development of advanced lesions
Maksimovic et al. [20]	Ultrasound	NA	Classification of carotid stenosis according to NASCET	Symptomatic	Patients with and without abdominal obesity did not significantly differ, either in the degree of carotid stenosis or in the degree of its clinical manifestation
Galarza-Delgado et al. [21]	Ultrasound	Focal structure that invades the lumen of the artery by at least 0.5 mm or 50% of the value of intima-media thickness, or when the thickness is equal to or greater than 1.5 mm when measured from the adventitia-media interphase to the intima-arterial lumen interphase	NA	NA	Presence of plaque was associated with abdominal circumference
Cuspidi et al. [22]	Ultrasound	NA	NA	Asymptomatic	No association was found between abdominal obesity and carotid plaque
Chiquete et al. [23]	US doppler	NA	Classification of carotid stenosis according to NASCET	Symptomatic (30 patients) Asymptomatic (503 patients)	There was no association between obesity and carotid stenosis ≥ 50% or between obesity and symptomatic carotid stenosis
Irie et al. [24]	Ultrasound	Focal structure encroaching into the arterial lumen or demonstrating a thickness >1.0 mm as measured from the media–adventitia interface to the intra-lumen interface	NA	Asymptomatic	The presence of echolucent carotid plaques with low gray-scale median values was related to high BMI
Yan et al. [25]	US doppler	Focal encroachment of internal carotid artery walls on either side	NA	Asymptomatic	There was no significant association between abdominal obesity or overweight/obesity with carotid plaques
Yuan et al. [26]	CT scan	Calcium mass score	NA	NA	No association was found between BMI, WC, and VAT, SAT determined on CT scan and carotid calcification
Pan et al. [27]	Ultrasound	NA	NA	NA	In females, the prevalence of carotid atherosclerosis was significantly higher in obese than in the control group
Radmard et al. [28]	Ultrasound	Localized thickening of >1.2 mm, not involving the whole circumference of the artery	NA	NA	Subjects with the highest amount of VAT were more prone to have more than one carotid plaque in comparison with participants showing the highest values of SAT or other conventional anthropometric indices
Sandfort et al. [29]	Magnetic resonance	NA	Total wall volume measurements	NA	Obesity was associated with the progression of carotid atherosclerosis in a low- to moderate-risk population treated with optimal statin therapy
Mitevska et al. [30]	US doppler	Detection of an IMT > 1.3 mm or a focal structure emerging from the wall of at least 0.5 mm or 50% of the surrounding IMT value	Carotid stenosis greater than 60% was considered significant	Asymptomatic	Multivariate analysis showed that obesity was not an independent predictor for the presence of carotid plaques
Higuchi et al. [31]	US doppler	NA	Stenosis was regarded as significant if stenosis rate ≥70	Asymptomatic	Visceral fat ≥ 100 cm^2^ was independently associated with cervical plaque. BMI and WHR were not
Mancusi et al. [32]	Ultrasound	IMT ≥ 1.5 mm	NA	Asymptomatic	Obesity was associated with a modestly increased prevalence of carotid plaques
Nishizawa et al. [33]	Autopsy	The largest atheroma plaque in the carotid artery was determined to calculate the stenosis index	The stenosis index was calculated by subtracting the lumen area from the outer area, dividing the difference by the outer area, and multiplying the result by 100	NA	Visceral fat was not associated with carotid artery stenosis index
Omisore et al. [34]	Ultrasound	Plaque was defined as focal thickening of at least 50% greater than that of the surrounding vessel wall, with a minimum thickness of at least 1.5 mm	NA	NA	Carotid plaques were associated with obesity
Imahori et al. [35]	Ultrasound	Localized protrusion of the vessel wall into the lumen of at least 50% compared with the adjacent intima-media thickness	NA	NA	BMI, WC, and WHtR were not associated with the presence of carotid plaques. The main measure of central obesity (WHR) showed the strongest and most consistent association with plaque presence and with plaque area
Laugesen et al. [36]	Magnetic resonance	NA	Carotid artery plaque burden was measured as maximum wall thickness derived from the lumen area and total vessel area outlines, maximum wall area, and maximum normalized wall index	Asymptomatic	Obesity was associated with increased carotid plaque necrotic core volume and calcification
Yoshida et al. [37]	Ultrasound	Localized elevated lesions with a maximum thickness of more than 1 mm	NA	NA	Visceral adiposity is an independent predictor of atherosclerosis
Scicali et al. [38]	Ultrasound	IMT greater than 1.5 mm	NA	Asymptomatic	The presence of carotid plaque was associated with high WHR
Rovella et al. [4]	Histology	Collected at carotid endarterectomy	NA	Symptomatic (265 patients) Asymptomatic (125 patients)	Obesity is an independent risk factor for carotid plaque destabilization
Geraci et al. [39]	US doppler	Focal structure encroaching into the arterial lumen of at least 0.5 mm or 50% of the surrounding carotid IMT value or carotid IMT > 1.5 mm	NA	Asymptomatic	ABSI was the only anthropometric adiposity index independently associated with the presence of carotid atherosclerotic plaque
Haberka et al. [40]	US doppler	Presence of plaques in the common carotid artery, bulb, and internal carotid artery	Classification of carotid stenosis according to NASCET	NA	None of the obesity measurements revealed an association with carotid atherosclerosis severity

## Abbreviations

BMI—body mass index; BSA—body surface area BRI—body roundness index; ABSI—a body shape index; CT scan—computerized tomography scan; DXA—Dual-energy X-ray absorptiometry; HC—hip circumference; IMT—intima-media thickness; MACE—major adverse cardiac events; MINORS—methodological index for non-randomized studies; MRI—magnetic resonance imaging; NA—not applicable; NASCET—North American symptomatic carotid endarterectomy trial; VAT—visceral adipose tissue; SAT—subcutaneous adipose tissue; WC—waist circumference; WHR—waist-to-hip ratio; WHtR—waist-to-height ratio.
